# Cyclin D1 expression is correlated with cell differentiation and cell proliferation in oral squamous cell carcinomas

**DOI:** 10.3892/ol.2014.1880

**Published:** 2014-02-13

**Authors:** YUICHI OHNISHI, MASAHIRO WATANABE, MASAHIRO WATO, AKIO TANAKA, KENJI KAKUDO, MASAMI NOZAKI

**Affiliations:** 1 Second Department of Oral and Maxillofacial Surgery, Osaka Dental University, Hirakata, Osaka 573-1121, Japan; 2 Department of Oral Pathology, Osaka Dental University, Hirakata, Osaka 573-1121, Japan; 3 Department of Cell Biology, Research Institute for Microbial Diseases, Osaka University, Suita, Osaka 565-0871, Japan

**Keywords:** oral squamous cell carcinoma, cyclin D1, Ki-67, differentiation, metastasis, pre-operative adjuvant therapy

## Abstract

The present study conducted an immunohistochemical investigation of cyclin D1 and Ki-67 expression in oral squamous cell carcinoma (SCC) to evaluate the correlations between cell differentiation, cell proliferation and metastasis, and the effect of anticancer drug medication and cyclin D1 expression. Cyclin D1 and Ki-67 were detected clearly in the nuclei of 35 SCC samples. No correlation between cyclin D1 protein expression and oral SCC differentiation was found. By contrast, the majority of metastatic foci (90%) exhibited strong cyclin D1 expression, whereas weak expression was observed in metastatic foci with pre-operative adjuvant therapy. Additionally, cyclin D1 and Ki-67 were expressed in basal to suprabasal cells of well-differentiated oral SCC, whereas cyclin D1-positive and Ki-67-negative cells were present in the highly-differentiated region, according to a double-immunostaining method. These results indicate that the expression of cyclin D1 protein plays a role in cell differentiation and cell proliferation in well-differentiated oral SCC.

## Introduction

Squamous cell carcinoma (SCC) is the most frequent type of cancer in the oral and maxillofacial region, and its metastatic and invasive abilities result in a poor prognosis (1,2). Standard care for oral cancer includes a combination of surgery, radiation and chemotherapy. Although cancer treatment is progressing substantially, the survival rate of patients with oral cancer has not changed over the past 30 years (3). To develop a novel effective therapy for oral cancer, a further understanding of the processes and molecules that lead to the initiation and progression of oral cancer is required. Cancer proteins not only cause and generate cancer, but also contribute to continuous proliferation and cancer survival; therefore, the proteins are considered useful therapeutic targets.

Cyclin D1 has been identified as a human oncogene (4). Rearrangement of the cyclin D1 gene locus, resulting in protein overexpression, has been associated with prognosis in a variety of malignant tumours, including oral SCC (5–9). Cyclin D1 is a protein with various functions, including cell cycle induction, transcription regulation and DNA damage-induced apoptosis (10,11). In oral SCC, there is a possibility that the expression of cyclin D1 may be associated with proliferation of a cancer cell, based on an association with Ki-67 expression (12). However, little evidence has been reported with regard to other possibilities or effects of cyclin D1.

The present study analysed the expression of cyclin D1 and Ki-67 using the immunohistochemical analysis of serial tissue sections and the double-staining method for samples of oral SCC.

## Materials and methods

### Patients

Between the years 2001 and 2011, 35 patients with operable oral cancer underwent surgery at the Department of Oral and Maxillofacial Surgery, Osaka Dental University Hospital (Osaka, Japan; Table I). The present study followed the tenets of the Declaration of Helsinki and was approved by the ethics committee of Osaka Dental University. Informed consent was obtained from the patients. None of the primary foci received pre-operative adjuvant therapy, and among 16 metastatic samples, 6 received pre-operative adjuvant therapy. The specific parameters of adjuvant therapy are shown in Table II. The histological classification of tumours was evaluated based on the Union for International Cancer Control (UICC) classification (13).

### Immunohistochemistry

Tissue samples obtained from patients with different stages of oral cancer were immediately fixed in 10% neutral buffered formalin solution (Sumitani Ind Ltd, Tottori, Japan) subsequent to resection and then embedded in paraffin (Thermo Fisher Scientific, Waltham, MA, USA). Sections (4-μm thick) were cut and mounted onto silane-coated glass slides (Matsunami Glass Ind Ltd., Osaka, Japan). Sections were deparaffinised in L-limonene (Falma Co., Ltd., Tokyo, Japan) and dehydrated through a graded ethanol series. Antigen retrieval was performed by autoclaving at 121°C for 15 min in Tris-EDTA buffer (pH 7.0). Endogenous peroxidase activity was blocked with 3% H_2_O_2_ for 10 min, and non-specific reactions were blocked by incubation with blocking solution (Nacalai Tesque, Kyoto, Japan) for 10 min. The tissue sections were incubated with a rabbit anti-cyclin D1 monoclonal antibody (1:500; Dako, Tokyo, Japan) or mouse anti-Ki-67 monoclonal antibody (1:100; Dako) at room temperature for 1 h. The tissue slides were then incubated with peroxidase micropolymer-conjugated secondary antibodies (Vector Laboratories, Burlingame, CA, USA) at room temperature for 30 min and visualised by incubation with a 3,3′-diaminobenzidine tetrahydrochroride liquid system (Dako) at room temperature for 5 min. The sections were then counterstained with hematoxylin (Merck KGaA, Daarmstadt, Germany) and observed by light microscopy (BX50, Olympus Corporation, Tokyo, Japan).

For double-immunostaining, the tissue sections were incubated with rabbit anti-cyclin D1 monoclonal antibody (1:100; Dako) overnight at 4°C. The tissue slides were then incubated with alkaline phosphatase-conjugated anti-rabbit immunoglobulin G (IgG) (Vector Laboratories) at room temperature for 30 min and visualised with PermaRed (Diagnostic Biosystems, Pleasanton, CA, USA). Antigen inactivation was performed by incubation at 98°C for 20 min in citrate buffer (pH 6.0). The tissue sections were incubated with mouse anti-Ki-67 monoclonal antibody (1:100; Dako) overnight at 4°C, and then the tissue slides were incubated with alkaline phosphatase-conjugated anti-mouse IgG (Vector Laboratories) at room temperature for 30 min and visualised with PermaBlue (Diagnostic Biosystems). The sections were then observed by light microscopy (Olympus Corporation).

### Evaluation of slides

The immunoreactivity of the cyclin D1 and Ki-67 proteins was evaluated by two independent pathologists with no knowledge of the patients’ clinicopathological factors and outcomes. The nuclear expression of the cyclin D1 and Ki-67 proteins was scored semi-quantitatively by combination of the staining intensity (scored as: 1, weak staining; 2, moderate staining; and 3, strong staining) and the proportion of positively-stained tumour cells in 1,000 tumour cells per high-power field (scored as: 0, <20%; 1, 20–40%; 2, 41–60%; 3, 61–80%; and 4, >80%). The sum of the staining intensity scores and the percentage of positive tumour cell scores were graded as follows: +, 1–3; ++, 4–5; and +++, 6–7. There was no discrepancy in the overall interpretation of the immunohistochemistry results between the two independent pathologists.

### Statistical analysis

A Mann-Whitney U test was performed using the SPSS software package (version 13.0, SPSS, Inc., Chicago, IL, USA) to assess statistically significant differences between samples. Data are presented as the means ± SD. P<0.05 was considered to indicate a statistically significant difference.

## Results

**Figure 1:**
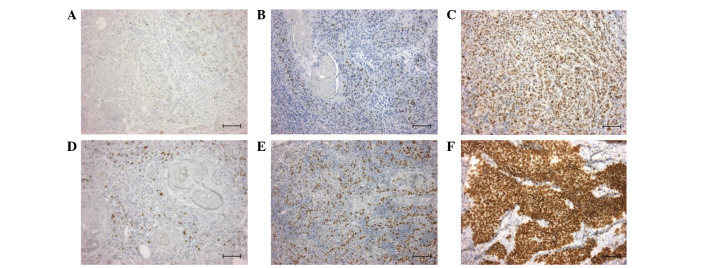
Expression levels of cyclin D1 and Ki-67 in representative oral SCC sections by immunohistochemical staining. (A) Weak (+), (B) moderate (++) and (C) strong (+++) expression levels of cyclin D1 in oral SCC tissues. (D) Weak (+), (E) moderate (++) and (F) strong (+++) expression levels of Ki-67 in oral SCC tissues. Scale bars, 100 μm. SCC, squamous cell carcinoma.

**Figure 2:**
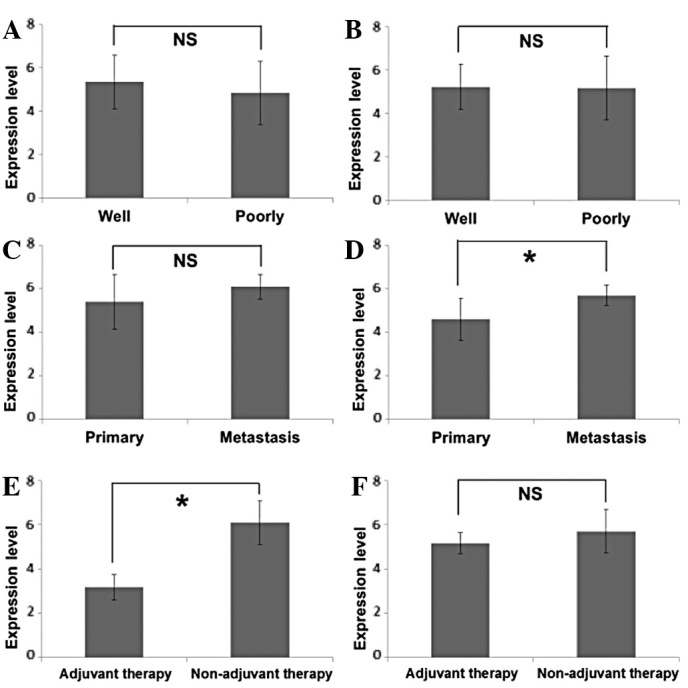
Cyclin D1 and Ki-67 expression in oral SCC. (A) Cyclin D1 and (B) Ki-67 expression levels differ between well- and poorly-differentiated primary oral SCC foci. Difference in (C) cyclin D1 and (D) Ki-67 expression levels between primary and metastatic foci in oral SCC. Difference in (E) cyclin D1 and (F) Ki-67 expression levels between metastatic oral SCC foci of patients with or without pre-operative adjuvant therapy. Data are presented as the mean ± SD (Mann-Whitney U test; ^*^P<0.05). NS, no significance; SD, standard deviation; SCC, squamous cell carcinoma.

Immunohistochemical staining was performed to investigate the expression of cyclin D1 and Ki-67 proteins in oral SCC clinical samples. Cyclin D1 and Ki-67 proteins were detected in the nuclei of the cells at various levels in all 35 samples examined. Cyclin D1 expression was observed in 4 cases with weak expression (+; Fig. 1A), 16 cases with moderate expression (++; Fig. 1B) and 15 cases with strong expression (+++; Fig. 1C) in primary foci (Table III). Ki-67 was observed in 2 cases with weak expression (+; Fig. 1D), 17 cases with moderate expression (++; Fig. 1E) and 16 cases with strong expression (+++; Fig. 1F) in primary foci (Table III). No correlation was found between the overexpression of cyclin D1 and Ki-67, and gender, region, tumour status or nodal status (Table III). No difference was observed in the expression levels of cyclin D1 and Ki-67 between poorly- and well-differentiated SCC (Figs. 2A and B). A statistical difference in cyclin D1 expression was not identified between primary foci and metastatic foci (Fig. 2C), but it should be noted that 90% of metastatic foci (9/10) showed strong cyclin D1 expression, while 43% (15/35) of primary foci demonstrated strong cyclin D1 expression (Table III). Ki-67 expression was significantly higher in metastatic foci than in primary foci (Fig. 2D). These results indicate a correlation between high levels of cyclin D1 expression and active cell proliferation of metastatic foci.

**Figure 3:**
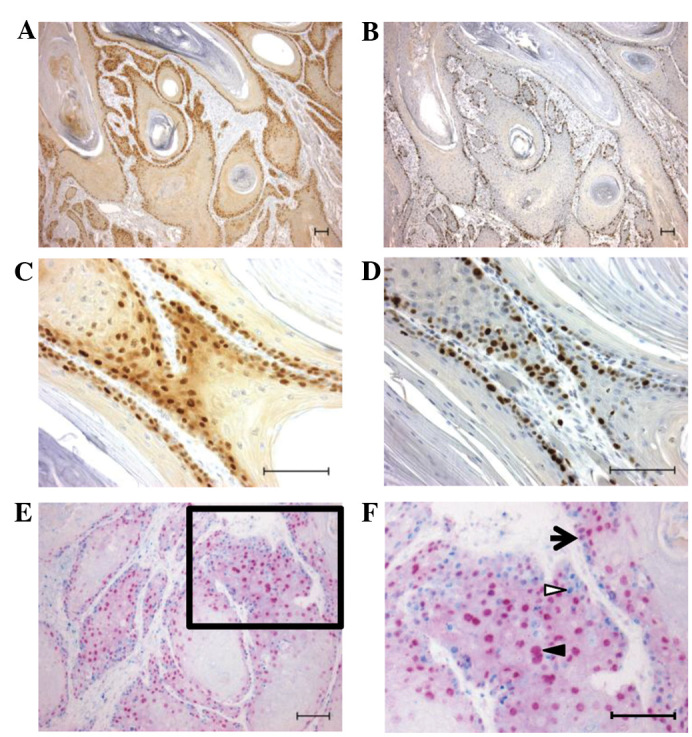
Expression of cyclin D1 and Ki-67 in oral SCC samples by immunohistochemical staining. (A and C) Expression of cyclin D1 and (B and D) Ki-67 in oral SCC tissues. (E and F) Immunohistochemical double-staining of cyclin D1 and Ki-67 in oral SCC. Cyclin D1-positive cells are represented in red (black arrowhead), Ki-67-positive cells are represented in blue (white arrowhead) and double positive cells are represented in purple (arrow). Scale bars, 100 μm. SCC, squamous cell carcinoma.

Furthermore, although lower levels of cyclin D1 expression were detected in metastatic foci with pre-operative adjuvant therapy (Fig. 2E), no effect was observed in Ki-67 (Fig. 2F). To date, the high expression of cyclin D1 in cancer tissue has been believed to play a role in cell proliferation due to its correlation with Ki-67 expression (12). To confirm the simultaneous occurrence of cyclin D1 and Ki-67 expression in the present study, immunohistochemistry was applied to serial tissue sections, and it was identified that cyclin D1 (Fig. 3A and C) and Ki-67 (Fig. 3B and D) were expressed in the basal to suprabasal cells.

In addition, the expression of cyclin D1 and Ki-67 was analysed using a double-immunostaining method in the same tissue sections. Cells expressing cyclin D1, but not Ki-67, were found to be located away from the basal cell layer (Fig. 3E and F).

## Discussion

Immunohistochemical staining was performed to investigate the expression of cyclin D1 in oral SCC. Cyclin D1 protein was detected in the nuclei of cells in all 35 samples examined. The expression level did not correlate with tumour differentiation, but it was higher in the metastatic foci than in the primary foci. Furthermore, there was a low level of expression in the metastatic foci with pre-operative adjuvant therapy. Ki-67 protein was also detected in all the samples investigated, with specific differences in expression levels. These results are fundamentally consistent with previous studies (12,14), in which cyclin D1 and Ki-67 expression was detected in the same region of oral SCC. Other studies have reported contradictory reports with regard to cyclin D1 expression, with certain studies indicating high expression of cyclin D1 in poorly-differentiated SCC (15,16), and others reporting the opposite, namely, high expression in well-differentiated SCC (17). However, these studies also reported rather low rates of cyclin D1-positive tumours compared with the data of the present study; potential bias caused by the low positive rate could partially explain these discrepancies.

In the present study, the expression of cyclin D1 and Ki-67 was examined in detail using the double-immunostaining method. Co-expression of cyclin D1 and Ki-67 was observed in the basal to suprabasal cells, and numerous cyclin D1-positive and Ki-67-negative cells existed toward the central section of the tumour. In well-differentiated oral SCC, poorly-differentiated and highly-proliferative cells that express Ki-67 are located in the basal layer, and proliferation slows down as it moves from the periphery to the centre of a tumour tissue and is occupied by differentiated cells that express keratin 17 (18). Therefore, the results of the present study indicate that the high expression of cyclin D1 observed in the cells was involved in the process of differentiation. Cyclin D1 in the nucleus is believed to promote the cell cycle by regulating the G_1_/S transition through an interaction with cyclin dependent kinase (CDK)2/4 (19). If cyclin D1 is highly expressed and the complex level of CDK2/4 increases, it not only promotes proliferation, but also reduces cell differentiation without entering into the G_0_ phase (20). Additionally, when cyclin D1 works with proteins other than CDKs, it may control apoptosis, aging, invasion and other processes through transcription or the DNA damage response (11,21–25). Therefore, in oral SCC, it is possible that cyclin D1 is involved in cell differentiation and the prevention of cell death, in addition to the cell proliferation that has been observed when working with proteins other than CDKs.

Lower levels of cyclin D1 expression were also found in the present study in the metastatic foci of patients with pre-operative adjuvant therapy compared with patients who did not receive pre-operative adjuvant therapy. By contrast, Ki-67 levels did not differ between the two groups of patients. The high expression of cyclin D1 may contribute to drug resistance in cancer cells, not only by increasing cell proliferation, but also by suppressing cancer cell apoptosis (26). The results of the present study indicate that cancer cells with a high expression of cyclin D1 and with drug resistance may survive, even if certain tumour cells of primary foci die and local control occurs as the result of pre-operative adjuvant therapy. Therefore, it is believed to be necessary to use certain methods to aid in the reduction of cyclin D1 levels, with respect to conventional anticancer drug medication.

## Figures and Tables

**Table I:** Clinicopathological factors in 35 patients with OSCC.

Variable	Well-differentiated	Poorly-differentiated
Gender, n		
Male	10	10
Female	13	2
Age, years		
Mean	66.2	63.7
Range	39–82	47–77
Region, n		
Tongue	15	3
Gingiva	4	8
Oral cavity floor	0	1
Buccal mucosa	3	0
Palate	1	0
T status, n		
T1	8	1
T2	12	6
T3	3	2
T4	0	3
N status, n		
N0	13	6
N1	4	1
N2a	0	0
N2b	6	5
Pre-operative adjuvant therapy, n		
Yes	1	5
No	9	1

OSCC, oral squamous cell carcinoma; T, primary tumour; N, regional lymph nodes.

**Table II:** Pre-operative adjuvant therapy regimen.

Patient no.	Differentiation level	Regimen
1	Well-differentiated	PEP+CDDP+TS-1^®^+RT
2	Poorly-differentiated	PEP+RT
3	Poorly-differentiated	CDDP+5-FU
4	Poorly-differentiated	TS-1^®^+RT
5	Poorly-differentiated	PEP+RT
6	Poorly-differentiated	CDDP+5-FU+RT

PEP, pepleomycin; CDDP, cisplatin; 5-FU, 5-fluorouracil; TS-1^®^, tegafur-gimeracil-oteracil potassium; RT, radiation therapy.

**Table III:** Correlation of Cyclin D1 and Ki-67 expression with clinicopathological factors in 35 patients with OSCC.

	Expression of Cyclin D1				Expression of Ki-67			
				
Variable	+	++	+++	P-value	+	++	+++	P-value
Gender, n								
Male	2	8	10	NS	0	12	8	NS
Female	2	8	5		2	5	8	
Region, n								
Tongue	2	5	11	NS	0	9	9	NS
Gingiva	2	7	3		2	5	5	
Oral cavity floor	0	1	0		0	0	1	
Buccal mucosa	0	2	1		0	2	1	
Palate	0	1	0		0	1	0	
T status, n								
T1	0	5	4	NS	0	6	3	NS
T2	3	8	7		2	9	7	
T3	1	1	3		0	1	4	
T4	0	2	1		0	1	2	
N status, n								
N1	0	1	3	NS	0	1	3	NS
N2a	0	0	0		0	0	0	
N2b	0	0	6		0	2	4	
N3	0	0	0		0	0	0	
Primary foci, n								
Well-differentiated	2	11	10	NS	1	12	10	NS
Poorly-differentiated	2	5	5		1	5	6	
Metastasic foci, n								
Well-differentiated	0	1	8	NS	0	3	6	NS
Poorly-differentiated	0	0	1		0	0	1	
Adjuvant therapy (metastasis), n								
Yes	3	3	0	<0.01	0	3	3	NS
No	0	1	9		0	3	7	

OSCC, oral squamous cell carcinoma; NS, no significance; T, primary tumour; N, regional lymph nodes.
